# Body size estimation of self and others in females varying in BMI

**DOI:** 10.1371/journal.pone.0192152

**Published:** 2018-02-09

**Authors:** Anne Thaler, Michael N. Geuss, Simone C. Mölbert, Katrin E. Giel, Stephan Streuber, Javier Romero, Michael J. Black, Betty J. Mohler

**Affiliations:** 1 Max Planck Institute for Biological Cybernetics, Tübingen, Germany; 2 Centre for Integrative Neuroscience, Tübingen, Germany; 3 Max Planck Institute for Intelligent Systems, Tübingen, Germany; 4 Graduate Training Centre of Neuroscience, International Max Planck Research School, University of Tübingen, Tübingen, Germany; 5 Department of Psychosomatic Medicine, University of Tübingen, Tübingen, Germany; Universita degli Studi di Udine, ITALY

## Abstract

Previous literature suggests that a disturbed ability to accurately identify own body size may contribute to overweight. Here, we investigated the influence of personal body size, indexed by body mass index (BMI), on body size estimation in a non-clinical population of females varying in BMI. We attempted to disentangle general biases in body size estimates and attitudinal influences by manipulating whether participants believed the body stimuli (personalized avatars with realistic weight variations) represented their own body or that of another person. Our results show that the accuracy of own body size estimation is predicted by personal BMI, such that participants with lower BMI underestimated their body size and participants with higher BMI overestimated their body size. Further, participants with higher BMI were less likely to notice the same percentage of weight gain than participants with lower BMI. Importantly, these results were only apparent when participants were judging a virtual body that was their own identity (Experiment 1), but not when they estimated the size of a body with another identity and the same underlying body shape (Experiment 2a). The different influences of BMI on accuracy of body size estimation and sensitivity to weight change for self and other identity suggests that effects of BMI on visual body size estimation are self-specific and not generalizable to other bodies.

## 1 Introduction

In the last decades, the mean body mass index (BMI) has significantly increased worldwide. Between 1975 and 2014, obesity prevalence has more than doubled, resulting in 10.8% of men and 14.9% of women being obese [1]. According to the international classification of body weight [2], obesity is defined as a BMI of 30 and above, overweight as a BMI between 25 and 29.9, normal weight as a BMI between 18.5 and 24.9, and underweight as a BMI below 18.5. Excess body weight is associated with a variety of diseases, such as cardiovascular diseases and diabetes, and can reduce life expectancy by up to eight years [3]. In line with the Health Belief Model [4], it has been suggested that a disturbed ability to accurately identify own weight and/or changes in own weight may contribute to overweight [5–7]. The goal of the current research was to test whether one’s personal body size predicts the accuracy of estimating own body size, the sensitivity to weight changes, and the desired body weight. By comparing body size estimation (BSE) of self and another identity, we additionally addressed whether distortions in BSE were due to a general bias in estimating body size, or were self-specific, and further whether they could be explained by cognitive-affective factors.

There are multiple body representations that are informed by different modalities and that determine how the own body and its size are perceived [8]. A recently suggested framework argues that the different body representations can be arranged on a continuous axis depending on how implicit versus explicit they are, and thus how easily they can be accessed by conscious introspection [9]. Implicit representations are thought to be informed by proprioception, somatosensation, and interoception, whereas explicit representations are constructed based on visual information about the body and cognitive-affective factors. Using measures to assess implicit types of body representations, studies have observed that individuals with overweight and obesity have lower interoceptive sensitivity as indicated by difficulties in heartbeat detection [10], and have inaccurate size estimation of objects touching the skin in tactile size estimation tasks [11,12], potentially reflecting a disturbed sense of own body size. In another study however, these observations have not been replicated [13], such that it is still unclear whether implicit body representations are really disturbed in obesity.

Individuals with overweight and obesity were also shown to inaccurately estimate own body size when using measures to assess explicit body representations. Several studies found that individuals with overweight and obesity underestimate their own weight [14,15] and underrated their weight status in terms of BMI categories [16–18]. A potential explanation for this inaccurate estimation and categorisation of own body size has been, that estimates of weight category are calibrated by the bodies in our environment. Specifically, it has been suggested that increased exposure to high BMI bodies can lead to misperceptions of the weight status of individuals with overweight and obesity [19,20]. Further, similar biases have been found when classifying other people varying in BMI into a discrete number of weight categories (underweight, healthy weight, overweight, and obese). For example, Oldham and Robinson [19] found that participants underestimated the weight status of individuals with overweight and obesity as compared to the category they belonged based on objective measurements of height and weight. Given the increase in obesity prevalence and the role of social comparison in establishing societal weight norms, it has been suggested that a possible reason for the inability to correctly perceive weight status might be an adjustment of the visual reference of what constitutes a ‘normal’ weight [20,21]. Thus, failures in recognizing own and others’ overweight could result from comparing the bodies to the adapted ‘normal’ body size reference rather than from a misrepresentation of the own body.

The importance of a body size reference has also been emphasized by recent studies using depictive BSE tasks to investigate BSE in anorexia nervosa (AN), a population that is typically observed to overestimate own body size [22,23]. According to that, depictive methods assess explicit body representations of what people think their body looks like. For example, Cornelissen et al. [24] reanalysed the data of a previous study by Tovée et al. [25] in which AN patients and healthy participants estimated their body size by adjusting the size of body parts in a picture of themselves presented on a computer screen. They found that AN patients and healthy low BMI participants overestimated their body size and healthy high BMI participants underestimated their body size, a pattern predicted by a perceptual error in magnitude estimation called ‘contraction bias’. A contraction bias arises when a standard reference is used against which the size of other examples in that category are estimated. Accordingly, magnitudes larger than the reference are underestimated, and magnitudes smaller than the reference are overestimated. Similar results were found when patients with AN were presented with non-personalized 3D images of female bodies varying in BMI and responded to whether the presented body was ‘smaller’ or ‘larger’ than the own actual body [26], or when participants from the normal population estimated the weight of a body by moving a slider on a linear scale [27]. Further, Cornelissen and colleagues suggested that another perceptual phenomenon described by Weber’s law could contribute to the difficulty to detect increases in body size in people with overweight and obesity. Weber’s law describes that the just noticeable difference between two stimuli is proportional to their magnitude [28]. Thus, in order to recognize weight gain, high BMI individuals would need to gain more absolute weight than low BMI individuals.

Although previous studies assume that similar biases might underlie the estimation of own and other’s body size [26,27], none of the studies has systematically investigated this. Previous studies typically either assessed size estimation of one’s own body or others’ bodies, and even self-BSE was usually operationalized through comparison with a body of another identity or a reference body weight category. Bodies naturally differ a lot in shape and since shape cues are highly relevant for estimating body size, being able to directly compare BSE of self and other requires control over shape differences and the amount of exposure to the body shape. Further, previous studies often lacked body stimuli with realistic weight manipulations, e.g. by stretching or compressing photographs or digital images, e.g. [29,30]. Weight changes however are highly non-linear and happen mostly around the midsection of the body; manipulating images in the horizontal dimension creates especially unrealistic hip and shoulder regions [26]. Therefore, in this study we present a novel method that allows investigating estimation of and sensitivity to changes in both own and other weight; as well as novel stimuli that portray realistic statistically probable weight manipulations of personalized stimuli. More specifically, we used personalized virtual 3D bodies (avatars) that were generated based on body scans of each participant and that allowed realistic alterations of identity and body weight based on a statistical body model. Realistically manipulated weight changes of the avatars ensured that body size estimates reflected subjectively perceived body size without being influenced by effects of unrealistic body shape deformations. Further, we were able to investigate the influence of identity on BSE by altering the texture map of the body (own vs. other), thereby creating a person with own and another identity, while keeping the underlying body shape identical. The avatars were presented in an ecologically valid scenario mimicking a situation of standing in front of a full-length mirror or standing in front of a life-size person. Two psychophysical methods were used to investigate the accuracy of BSE, and the sensitivity to weight changes of self and other identity.

## 2 Overview of current experiments

The current set of experiments investigated the relationship between personal body size, indexed by BMI, and the accuracy of BSE, the sensitivity to weight changes, and the ideal body weight of self and other in a non-clinical population of female individuals varying in BMI. Experiment 1 tested whether one’s personal body size predicts BSE of a body with own identity. A depictive BSE task was used to assess an explicit representation of one’s body. Following Moelbert et al. [23], depictive BSE tasks mainly recruit explicit representations, namely of how participants think their body looks like in terms of body weight. In two well-established psychophysical experiments, participants were presented with their personalized avatars varying in weight in a virtual environment and responded whether the body presented corresponded to their actual body size (one-interval forced choice paradigm with two response possibilities; 1AFC) and adjusted the avatar until it matched the size they perceived their actual body to be, as well as the size of their ideal (desired) body (Method of Adjustment; MoA). Additionally, to test whether distortions in BSE could be explained by cognitive-affective factors, measurements of self-esteem, eating behaviour, and attitudes towards own body shape and weight were obtained using several validated questionnaires.

To addressed whether distortions in BSE in Experiment 1 were due to a general bias in estimating body size or were self-specific, the influence of identity on BSE was assessed in Experiment 2a and 2b by altering the texture map of the body (own vs. other) while keeping the underlying body shape identical. Participants memorized a body of another identity (Experiment 2a) or their own (Experiment 2b) and then judged whether weight variations of that body were identical to the memorized body. Experiment 2b served to control for the differences in the experimental design used to assess BSE of self (Experiment 1) and other (Experiment 2a). If participants exhibited similar patterns in BSE as in Experiment 1 when they no longer identified with the body (Experiment 2a), then distortions in estimating body sizes could be attributed to general biases in BSE. However, if participants exhibited different patterns in BSE in Experiment 2a than in Experiment 1 and 2b, then distortions in BSE may be driven by factors that are specific to self, for example attitudinal factors.

## 3 Experiment 1: Self-body size estimation

**Fig 1:**
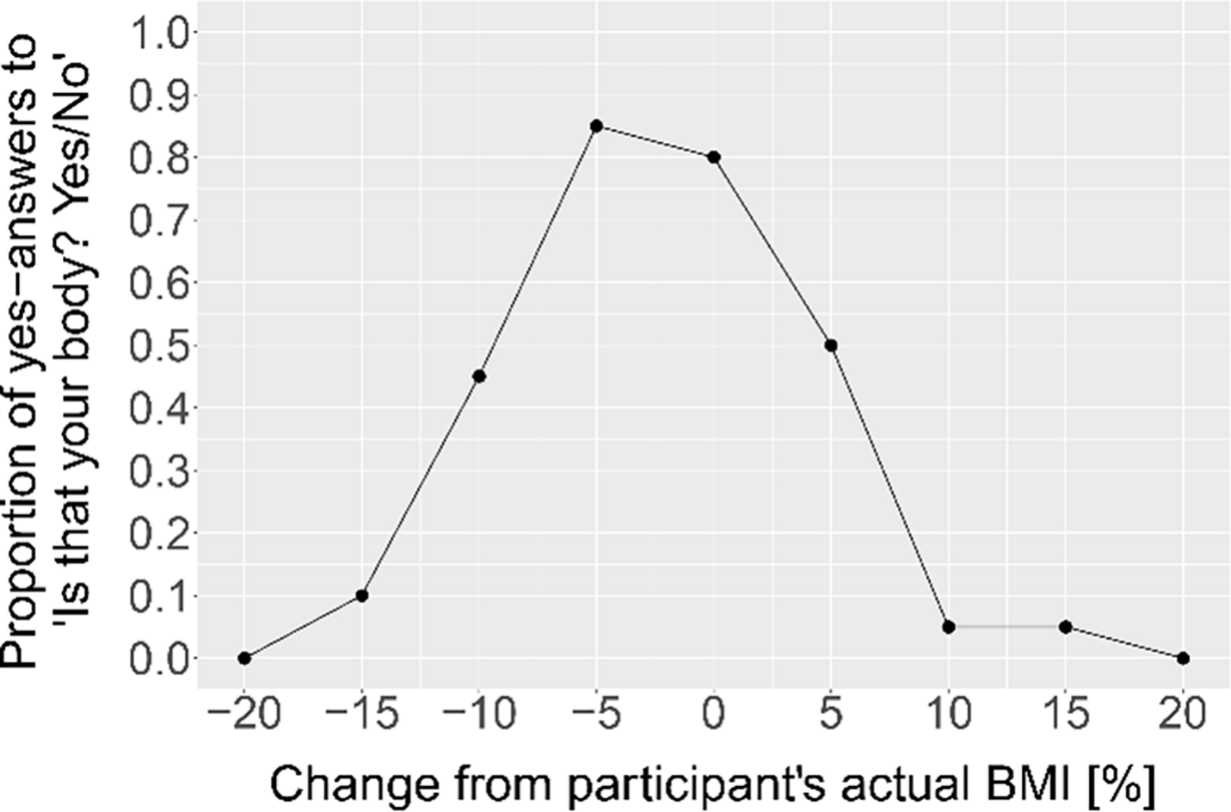
Example raw data of the 1AFC task of one participant representative of the group.

Experiment 1 investigated whether personal BMI predicts the accuracy of BSE as predicted by a contraction bias [26,31], and/or influences the sensitivity to positive and negative changes in BMI relative to participants’ estimated own body size. For each participant, personalized 3D bodies (avatars) with varying body weight were created. In the 1AFC task, participants judged whether the presented avatar (-20% to +20% of their actual BMI, at 5% intervals) corresponded to their actual body size or not by answering to the question ‘Is that your body? Yes/No’ (for example data, see Fig 1). The avatar with the highest proportion of yes-answers (peak) reflects each participant’s estimated body size. The percentage change of yes-answers indicates how sensitive participants were to positive and negative changes in BMI relative to their estimated body size. In the MoA task, participants adjusted the size of their personalized avatar until it matched the size of their actual body and their ideal body. The MoA task was employed in addition to the 1AFC task for two reasons. First, a smaller step size in the MoA task allowed a more precise measure of BSE than the peak in the 1AFC task. Second, the MoA task additionally provided a measure of each participant’s ideal body size and body dissatisfaction as represented by the discrepancy between estimated and ideal body size. The discrepancy between estimated and actual physical body size in both the 1AFC and MoA task was used as a measure of distortion of BSE. Further, several questionnaires were administered to assess cognitive-affective factors related to own body size to additionally examine whether those factors could explain distortions in BSE.

### 3.1 Method

#### 3.1.1 Participants

Fifty-four females were recruited (age in years: *m* = 26.54, *sd* = 7.11) based on BMI from the local community. Participants’ average BMI was 25.78 (*sd* = 6.84, BMI range: 16.94–43.89). According to the International Classification of body weight [2], 7.41% of the participants are classified as underweight (BMI < 18.5), 51.85% as normal weight (BMI 18.5–24.9), 16.67% as overweight (BMI 25–29.9), and 24.07% as obese (BMI ≥ 30). All participants had a high level of German proficiency, normal or corrected-to-normal vision, and no current or past eating or other psychiatric disorders as confirmed by the Structured Clinical Interview for DSM-IV (SCID-I, [32]). Descriptive statistics of all participants are shown in Table 1; for illustration purposes, participants were additionally split into the BMI weight categories. Participants gave written informed consent and were compensated with 8€ per hour for their participation. The experimental procedure was approved by the local ethics committee of the University of Tübingen, Germany and was in accordance with the Declaration of Helsinki. The individuals in this manuscript have given written informed consent (as outlined in PLOS consent form) to publish these case details.

**Table 1:** Descriptive statistics of the participants (n = 54).

	All participants (n = 54)		Underweight, BMI < 18.5 (n = 4),	Normal weight, BMI 18.5–24.9 (n = 28),	Overweight, BMI 25–29.9 (n = 9),	Obese, BMI ≥ 30 (n = 13),
	range	mean (sd)	mean (sd)	mean (sd)	mean (sd)	mean (sd)
Participant characteristics						
Height (m)	1.52–1.84	1.67 (0.07)	1.66 (0.06)	1.70 (0.07)	1.68 (0.06)	1.62 (0.06)
Weight (kg)	44.5–119.5	71.77 (16.87)	48.55 (3.47)	62.89 (6.52)	76.6 (8.12)	94.68 (12.82)
BMI (kg/m^2^)	16.94–43.89	25.78 (6.84)	17.72 (0.55)	21.69 (1.62)	27.23 (1.62)	36.09 (4.63)
Age (y)	18.00–55.00	26.54 (7.11)	26.00 (2.16)	24.25 (6.00)	27.22 (6.82)	31.15 (8.62)
Eating behaviour and body shape and size concerns						
Eating Disorder Inventory—Body Dissatisfaction	9.0–53.0	29.46 (11.40)	20.25 (1.71)	22.79 (7.56)	39.67 (10.44)	40.31 (6.99)
Eating Disorder Inventory—Drive for Thinness	7.0–40.0	16.91 (8.48)	14.25 (5.12)	12.64 (6.21)	23.56 (10.61)	22.31 (6.29)
Body Image—Negative Evaluation of the Body	11.0–50.0	22.94 (9.54)	20.25 (7.09)	17.14 (3.66)	29.11 (11.08)	32.0 (8.90)
Body Image—Perception of Body Dynamics	18.0–48.0	35.49 (6.72)	38.75 (7.54)	38.08 (5.18)	31.78 (7.73)	31.46 (6.15)
Rosenberg Self-Esteem Scale	9.0–30.0	22.34 (5.90)	22.00 (9.06)	24.11 (4.03)	20.78 (7.40)	19.73 (6.60)
Physical Appearance Comparison Scale	5.0–23.0	13.17 (3.54)	16.00 (4.32)	12.00 (3.44)	14.89 (3.98)	13.62 (2.29)

#### 3.1.2 Psychometric and biometric measures

*Clinical Interview and Psychometric Measures*. The Structured Clinical Interview for DSM-IV Axis I Disorders was used to confirm that none of the participants had any history of eating disorders, affective syndromes, addictions, anxiety disorders and somatic symptom disorders (SCID-I; [32]). For getting a measure of several psychometric variables, the validated German versions of the following questionnaires were used to assess self-esteem, eating behaviour, and emotional self-evaluation and attitudes towards own body size and shape: the Rosenberg self-esteem scale (R-SES; [33,34]), the subscales ‘body dissatisfaction’ (BD) and ‘drive for thinness’ (DT) of the Eating Disorder Inventory (EDI-2; [35]), as well as a German questionnaire for body image (BI) assessment [36] with the two subscales ‘vital body dynamics’ (VBD) and ‘negative body evaluation’ (NBE). Participants also completed the Physical Appearance Comparison Scale (PACS; [37]) which assesses habits of comparing own physical appearance to others.

*Anthropometric measurements*. Participants’ height and weight were measured using a stadiometer and a digital scale. BMI was calculated as weight/height^2^ [kg/m^2^].

#### 3.1.3 Stimuli and scene

For each participant, three body scans were collected using a 3D body scanner (3dMD, Atlanta/GA). The scanning system uses speckle projectors which project textured light patterns on the body, 22 stereo units composed of two black and white cameras observing the speckle pattern for recording the body geometry, and a 5-megapixel colour camera capturing the body texture. The system has a spatial resolution of approximately 1 mm. In order to get an accurate representation of the participants’ body shapes, they were asked to wear tight grey shorts and a sports bra. One participant wore a tight grey strapless top instead of the sports bra. To minimize distortions caused by the hair, participants wore a hair cap. Each participant was scanned in 3 different poses (T-pose, A-pose, and neutral) resulting in three high-polygon meshes and RGB images for texture generation. In order to generate the body stimuli, the meshes were first registered to a statistical body model as described by Hirshberg, Loper, Rachlin, and Black [38].

The statistical body model consists of a template mesh that can be deformed in shape and pose in order to fit a 3D-scan. The shape component of the body model was learned from 2,094 female bodies in the CAESAR dataset [39] by applying principal component analysis on the triangle deformations in the observed meshes after removing deformation due to pose. This allowed to model body shape variation in a subspace, *U*, spanned by the first 300 principal components, where the body shape of an individual, *S*_*j*_, is described as a vector of 300 linear coefficients, *β*_*j*_, that approximate the shape deformation as *S*_*j*_ = *Uβ*_*j*_ + *μ*, where *μ* is the mean shape deformation in the female population. The pose component of the body model compactly describes deformations due to body part rotations and is trained from approximately 1200 3D-scans of people in different poses. The registration process consists of the identification of pose and shape parameters that transform the template mesh into the scan by minimizing the distance between template mesh and scan. Once the scan is registered, a texture map is computed for the participant’s model based on the pixels from the 22 RGB calibrated images. The texture map was post-processed in Adobe Photoshop (CS6, 13.0.1) to conceal small artefacts and to standardize the colour of the textures across participants.

**Fig 2:**
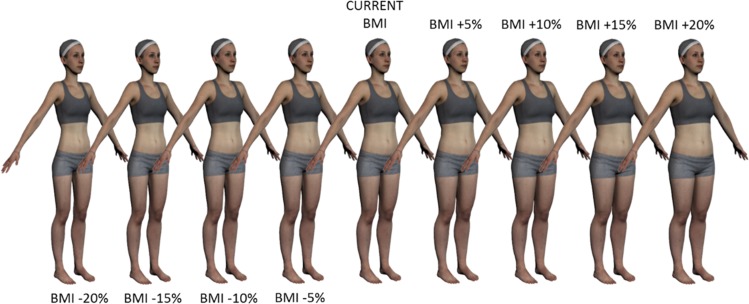
Set of personalized 3D bodies (avatars) with own shape and own photo-realistic texture. Note, the avatars in the experiment were always shown in front view.

In order to generate the different BMI versions of each avatar, a linear regressor X was learned between anthropomorphic measurements *A* = [weight, height, arm length, inseam] and the shape identity component *β* for the whole CAESAR dataset, so that the difference ||(*A*|1)*X*–*β*|| is minimized. This defines a linear relation between shape and measurements for each participant and allowed us to modify *β* in a way that produces intended changes in the anthropomorphic measurements. Given each participant’s weight *w*, height *h*, and registration, nine avatars were generated with varying BMIs $(1+\frac{\Delta BMI}{100})\frac{w}{h^{2}}$, where *ΔBMI* = {0, ±5%, ±10%, ±15%, ±20%} (Fig 2). Changing the BMI was achieved by applying a change in the shape vector, so that $\Delta \beta =\left[\frac{\Delta BMI}{100}w,0,0,0\right]∙X$ (i.e. changing the weight equally to the desired proportional change in BMI, while keeping the other measurements–height, arm length, inseam–constant (for more technical details see Piryankova et al. [40]).

In this study, all body stimuli were posed identically in an A-Pose (by keeping the pose parameters constant). An A-Pose was chosen to allow for weight changes without any influence on the angles of the body, such as the distance of the arms to the torso, or the thigh gap, as this would have provided additional cues for weight changes. The pose parameter vector was calculated as the average pose parameter vector of all registered scans in the A-pose. The pose was standardised to control for differences in participants’ pose and to put the focus on the shape of the body only. In addition, since the same body stimuli were used across all three experiments, we hypothesized that the manipulation of the avatar’s identity by swapping out the body texture (Experiment 2a) would have worked less well with keeping such a salient identification feature as individual body pose. For the MoA task, the nine personalized avatars were combined in Autodesk 3ds Max 2015, such that it was possible to morph between them in steps of 0.05% of the participant’s actual BMI.

**Fig 3:**
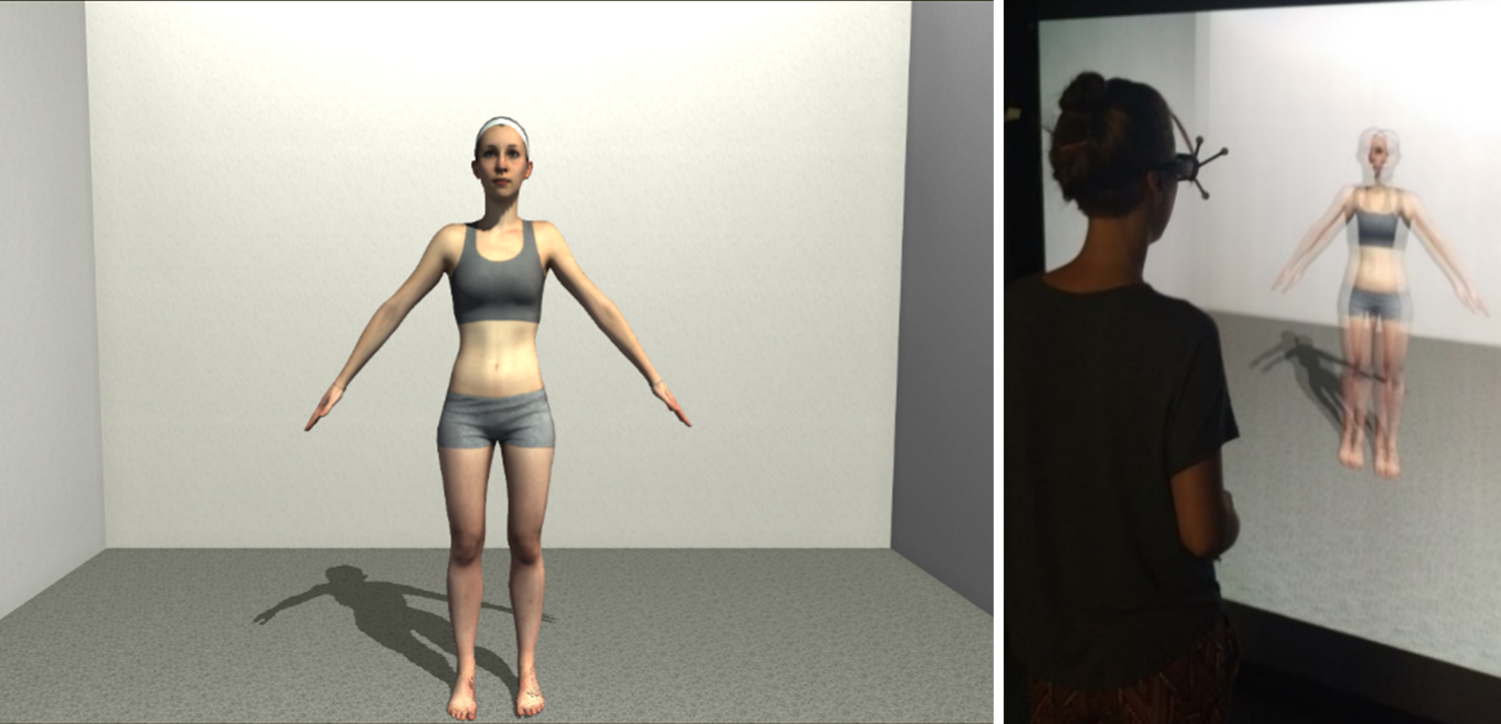
Left: Screenshot of the virtual scene viewed by the participants; Right: participant views personalized avatar on large-screen immersive stereo display.

During the experiment, participants stood 100 cm in front of a flat, large-screen immersive display on which the stimuli were projected using a back-projection system with a Christie SX+ stereoscopic video projector (1400 x 1050 native pixel resolution). The projected area covered 2.16 m × 1.62 m (94.4° x 78° of visual angle, horizontally x vertically) with a floor offset of 0.265 m. The stereoscopic projection was generated using an average interocular distance of 6.5 cm [41]. In order to see the scene stereoscopically, participants wore a pair of shutter glasses (nVidia 3D Vision Pro). The glasses had a field of view of 103° (horizontally) × 62° (vertically), corresponding to an area of 2.52 m × 1.2 m of the display. The display was connected to a motion tracking system (ART, SMARTTRACK) that tracked the position of reflective markers mounted onto the shutter glasses to provide motion parallax and thereby improve distance perception to the avatar and further to create natural viewing conditions where the visual perspective changes with head movements. The virtual scene was programmed in Unity 3D (Version 4.6.3f1, Unity Technologies) and contained a personalized virtual body standing in an empty virtual room at a distance of two meters from the participant (Fig 3, left). The avatars were presented in a constant A-Pose facing the participant and were mirror-inverted to mimic a situation as if the participant was looking at herself in a mirror. Participants responded by pressing buttons on a joystick pad.

#### 3.1.4 Procedure

The entire procedure was conducted across two sessions on different days. In the first session, demographic data was collected, and the clinical interview and the questionnaires were completed. Further, participants’ bodies were scanned and their body weight and height were measured. The whole session took between 45 and 90 minutes. Due to post-processing of the body scans, the second session took place on average 19.09 days (*sd* = 11.16) after the first session.

In the second session, participants first completed a one-alternative forced choice task with two response possibilities (1AFC) and then two blocks of a method of adjustment (MoA) task. The order of the two tasks was kept constant to ensure that the ability to interactively explore the full range of weight change in the MoA task would not influence the responses in the 1AFC task. Before the 1AFC task, participants were told that based on their body scan different versions of their body varying in body weight were created and their task would be to judge whether the body of the presented avatar corresponds to their own physical body. To avoid any influence of emotionally charged words (e.g. thinner, fatter) on body size estimates, participants were instructed to respond to the question ‘Is that your body? Yes/No’ via pressing buttons on a joystick pad. Participants were explicitly instructed that the question refers to the avatar’s body weight. Each of the nine personalized avatars with varying BMI (+0%, ±5%, ±10%, ±15%, and ±20% of the participant’s actual BMI) was shown 20 times, resulting in a total of 180 trials. The presentation of the nine avatars was blocked such that all nine avatars were viewed before being repeated. Within each block, the order of presentation was randomized. After every 45 trials, participants could take a break if needed. On each trial in the 1AFC task, participants saw one of the nine personalized avatars with own photo-realistic texture for 2000 milliseconds. Subsequently, the question ‘Is that your body? Yes/No’ was shown on a blue background and participants responded. Participants were encouraged to respond as accurately as possible, and there was no time limit placed on their response.

Following the 1AFC task, participants performed two blocks of a MoA task in which they first adjusted the size of the avatar nine times to match their actual body size and then nine times to match their ideal (desired) body size using buttons on a joystick pad. Participants could increase and decrease the body weight for an unlimited amount of times and pressed another button after they had finished adjusting the body. The initial avatar presented was one of the nine personalized avatars. The order of the initial avatars was randomized. Following the MoA tasks, participants completed a post-questionnaire assessing subjective similarity of the personalized avatar (that they thought would correspond to their actual body size) to their own physical body, with respect to overall impression, shape, appearance, arms, legs, torso, and face. Each item was rated on a 7-point Likert Scale from not similar at all (1) to very similar (7). In addition, participants’ body weight was measured to correct the body size estimates for weight changes between the body scan session and the experiment. The order of the tasks remained constant across participants. The whole session took around 45 minutes.

### 3.2 Statistical analysis

For each participant, the proportion of yes-answers to the question ‘Is that your body? Yes/No’ was calculated for each of the nine bodies in the 1AFC task (for an example, see Fig 1). The body with the highest proportion of yes-answers (peak) reflects the estimated own body size. To get a measure of the accuracy of BSE, the estimates were compared to the participants’ BMI at the time of the experiment. Further, since weight loss and weight gain are in general differently emotionally loaded, sensitivity to weight changes might depend on whether the presented body was bigger or smaller than the estimated own body size. Thus, the slopes on both sides of the peak were analysed separately. For each side (left, right) of each participant’s curve, a cumulative Weibull function was fit according to [42]. Alpha (position along the x-axis), beta (slope steepness), and lambda (peak) were free to vary. Gamma (flooring performance) was fixed to zero. As a measure for sensitivity to weight changes, the slope steepness (beta) was analysed. In the MoA tasks, the mean response of the nine trials was calculated for each participant per condition (actual body size, ideal body size). Due to a technical error discovered after data collection was finished, morphing in the MoA task did not always linearly occur when decreasing the avatar’s weight, but always correctly occurred when increasing the weight. Only in 3.27% of all trials, the final responses fell into the critical range and participants experienced this error in visual presentation of the body. Since participants were able to adjust the body weight for an unlimited amount of times both from above and below and appeared to be doing so as evident from examining the logged time courses of adjusting the avatar’s weight, we decided not to exclude these trials as this might have biased the results for people that responded in this range.

Linear regression analyses were used to test if participants’ BMI significantly predicted the peak and/or the slope steepness of the yes-answer distributions in the 1AFC task, and the estimated actual and ideal body size in the MoA tasks. The analyses were done in R, v 3.2.2, using the *lm* function (stats package).

### 3.3 Results

At the time of the experiment, the mean BMI of the participants was 25.94 (*SD* = 6.91) and had decreased on average by 0.28 BMI units (*SD* = 0.44; range: -1.30 to +0.82 BMI units) between the scan session and the experiment.

#### 3.3.1 Psychometric variables

To control for any influence of the psychometric variables (EDI-2-DT, EDI-2-BD, BI-VBD, BI-NBE, RSE, PACS) on the results of the psychophysical experiments, correlations between the questionnaire scores were calculated first using the *corr*.*test* function (psych package) in R v 3.2.2. Correlations are reported as Pearson correlation coefficients, and are corrected for multiple correlations using the Holm method [43]: EDI-2-DT and EDI-2-BD (*r* = 0.8, *p* < .001), EDI-2-DT and BI-VBD (*r* = -0.43, *p* < .01), EDI-2-DT and BI-NBE (*r* = 0.79, *p* < .001), EDI-2-DT and RSE (*r* = -0.47, *p* < .01), EDI-2-DT and PACS (*r* = 0.43, *p* < .01), EDI-2-BD and BI-VBD (*r* = -0.61, *p* < .001), EDI-2-BD and BI-NBE (*r* = 0.82, *p* < .001), EDI-2-BD and RSE (*r* = -0.51, *p* < .001), EDI-2-BD and PACS (*r* = 0.44, *p* < .01), BI-VBD and BI-NBE (*r* = -0.64, *p* < .001), BI-VBD and RSE (*r* = 0.68, *p* < .001), BI-VBD and PACS (*r* = -0.22, *p* = .11), BI-NBE and PACS (*r* = .42, *p* < .01), BI-NBE and RSE (*r* = - 0.65, *p* < .001), PACS and RSE (*r* = -0.41, *p* < .01). Since most of the psychometric variables were significantly correlated, a principal component analysis was used to determine the latent variable(s) in the psychometric data using the *psych* package. The Kaiser-Meyer-Olkin measure of sampling adequacy was .79, and thus above the recommended value of .5 [44]. The analysis revealed that the first three factors had Eigen values of 3.84, 0.84, and 0.70, and explained 63.98%, 13.97%, and 11.65% of the variance respectively. The scree plot showed that the point of inflexion occurred at the second factor thereby justified to retain only one factor. The factor loadings for EDI-2-DT, EDI-2-BD, BI-VBD, BI-NBE, RSE, and PACS were .70, .79, .57, .85, .33, and .60 respectively. The factor score from these latent variable, called PSY from here on, constitutes a combination of participants’ attitudes towards body weight and shape, eating behaviour and self-esteem. There was no significant correlation between PSY and participants’ BMI, *r* = .20, *t*(52) = 1.50, *p* = .14. PSY was used as a covariate in the regression models.

#### 3.3.2 1AFC

**Fig 4:**
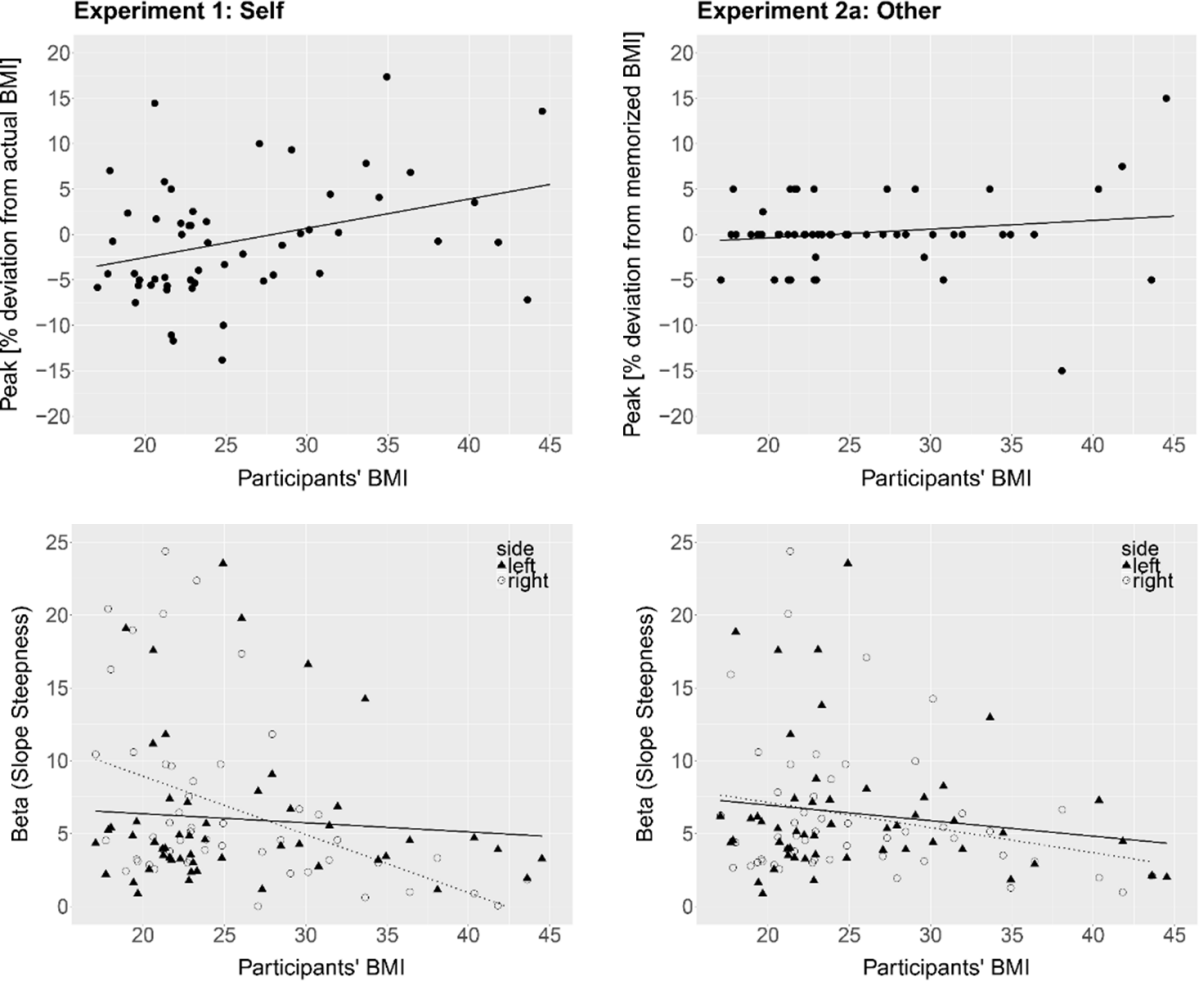
*Top*: Percent deviation of the estimated own body size (Experiment 1, *left*) and estimated memorized body size (Experiment 2a, *right*) from actual BMI (in %-BMI units) as a function of participants’ BMI in the 1AFC task. The lines represent regression lines. *Bottom*: Beta values (slope steepness of the yes-answer distribution) for presented bodies smaller (left side of the peak; triangles, solid line) and bigger (right side of the peak; circles, dotted line) than estimated own body size (Experiment 1, *left*) and estimated memorized body size (Experiment 2a, *right*) as a function of participants’ BMI in the 1AFC task. Higher beta values indicate greater sensitivity to %-BMI changes. The lines indicate the regression lines.

*Peak*. Estimated body size (body with the highest proportion of yes-answers; peak of yes-answer distribution), in raw units, was regressed onto participants’ BMI at the time of the experiment to test whether accuracy of BSE varied based on individual BMI. Participants’ actual BMI significantly predicted the accuracy of estimated body size in terms of percent of own BMI, B = .32, *t*(52) = 2.64, *p* = .01, *R*^*2*^ = .12 (Fig 4, top left). To control for influences of the psychometric variables, the PSY scores were included into the linear model. The two models were compared using an analysis of variance (ANOVA) to assess whether the amount of variance explained by the model significantly increased after adding in the PSY scores. PSY did not significantly increase the fit of the model, *F*(1,51) = 0.04, *p* = .85. Thus, the effect of BMI on the accuracy of BSE was not altered when controlling for differences in the psychometric variables.

*Slopes*. There was insufficient data to fit a Weibull function for three participants on the right side and one participant on the left side. For the remaining participants, the fit of the psychometric function to the data was good (mean *R*^*2*^ = .99, *sd* = .03; left side: mean *R*^*2*^ = .98, *sd* = .04; right side: mean *R*^*2*^ = .99, *sd* = .02). Since sensitivity to weight changes might depend on whether the presented bodies were bigger or smaller than the estimated own body size, linear regression analyses were used separately for the left and right slopes to test if participants’ BMI significantly predicted beta (slope steepness). BMI did not predict beta on the left side (B = -.06, *t*(51) = -0.61, *p* = .54, *R*^*2*^ = .01), but significantly predicted beta on the right side (B = -.40, *t*(49) = -3.32, *p* = .001, *R*^*2*^ = .18) (Fig 4, bottom left). Sensitivity to weight changes of bodies bigger than estimated own body size (right side of the peak) decreased as participants’ BMI increased. Within the context of the present experiment, sensitivity reflects the willingness to accept a given body as similar to one’s physical body. Thus, participants with a higher BMI were more willing to accept bodies bigger than their estimated body size as their own, as compared to participants with a lower BMI. Including the PSY variable into the linear models did not significantly increase the fit of the model on the right side, *F*(1, 48) = 1.49, *p* = .23, but significantly increased the fit on the left side, *F*(1, 50) = 6.88, *p* = .01. On the left side, there was a significant effect of PSY on beta values *t*(50) = 2.62, *p* = .01, but the effect of BMI remained non-significant, *t*(50) = -1.17, *p* = .25. Beta values increased with increasing PSY scores indicating that participants with a greater tendency for a disturbed emotional self-concept were more sensitive to bodies thinner than their estimated own body size.

#### 3.3.3 MoA

**Fig 5:**
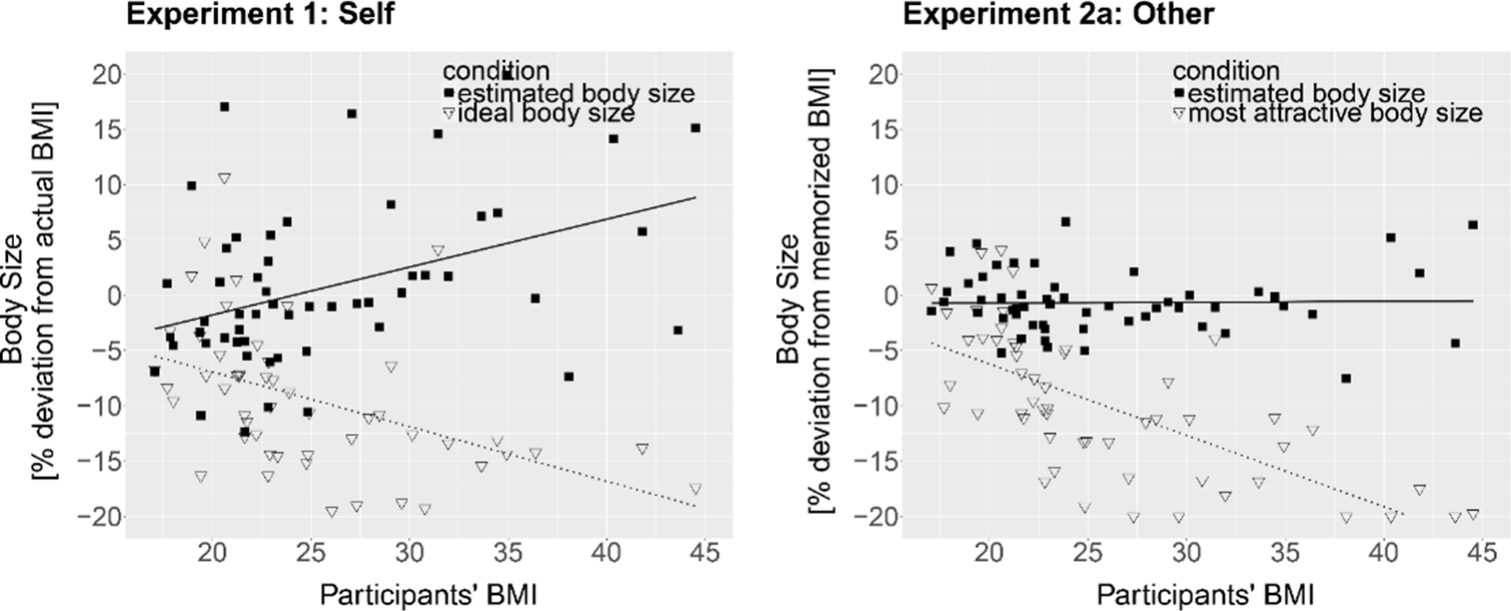
*Left*: BMI deviation [in %] of the estimated own body size (squares, solid line) and adjusted ideal body size (triangles, dotted line) as a function of participants’ BMI in the MoA task of Experiment 1. *Right*: BMI deviation [in %] of the estimated memorized body size (squares, solid line) and adjusted most attractive body size (triangles, dotted line) as a function of participants’ BMI in the MoA task of Experiment 2a. The lines represent regression lines.

Estimated body size, in raw units (% BMI change), was regressed onto participants’ BMI to examine whether the accuracy of BSE varied based on individual BMI. BMI significantly predicted the accuracy of estimated own body size (B = .43, *t*(52) = 3.23, *p* = .002, *R*^*2*^ = .17; Fig 5, left). As in the 1AFC task, participants’ body size estimates increased with increasing personal BMI such that lower BMI participants tended to underestimate their body size and higher BMI participants tended to overestimate their body size. Including the PSY scores into the regression model did not significantly increase the fit of the model, *F*(1,51) = 0.03, *p* = .88. Further, BMI significantly predicted raw scores of ideal body size (B = -.57, *t*(52) = -4.94, *p* < .001, *R*^*2*^ = .32; Fig 5, left). High BMI participants had a greater deviation between their actual and ideal BMI than low BMI participants. Participants’ ideal body size was lower than the estimated own body size for all participants except for three (participants’ BMI: 17.08, 17.85, and 19.6). Adding the PSY variable to the linear regression did not significantly increase the fit of the model, *F*(1,51) = 0.63, *p* = .45. In order to get a measure of body dissatisfaction, the difference between estimated and ideal body size was calculated. Raw scores of estimated/ideal BMI discrepancy were significantly predicted by BMI (B = .99, *t*(52) = 9.24, *p* < .001, *R*^*2*^ = .62). Discrepancy between estimated and ideal body size increased with increasing BMI.

#### 3.3.4 Possible confounds

**Fig 6:**
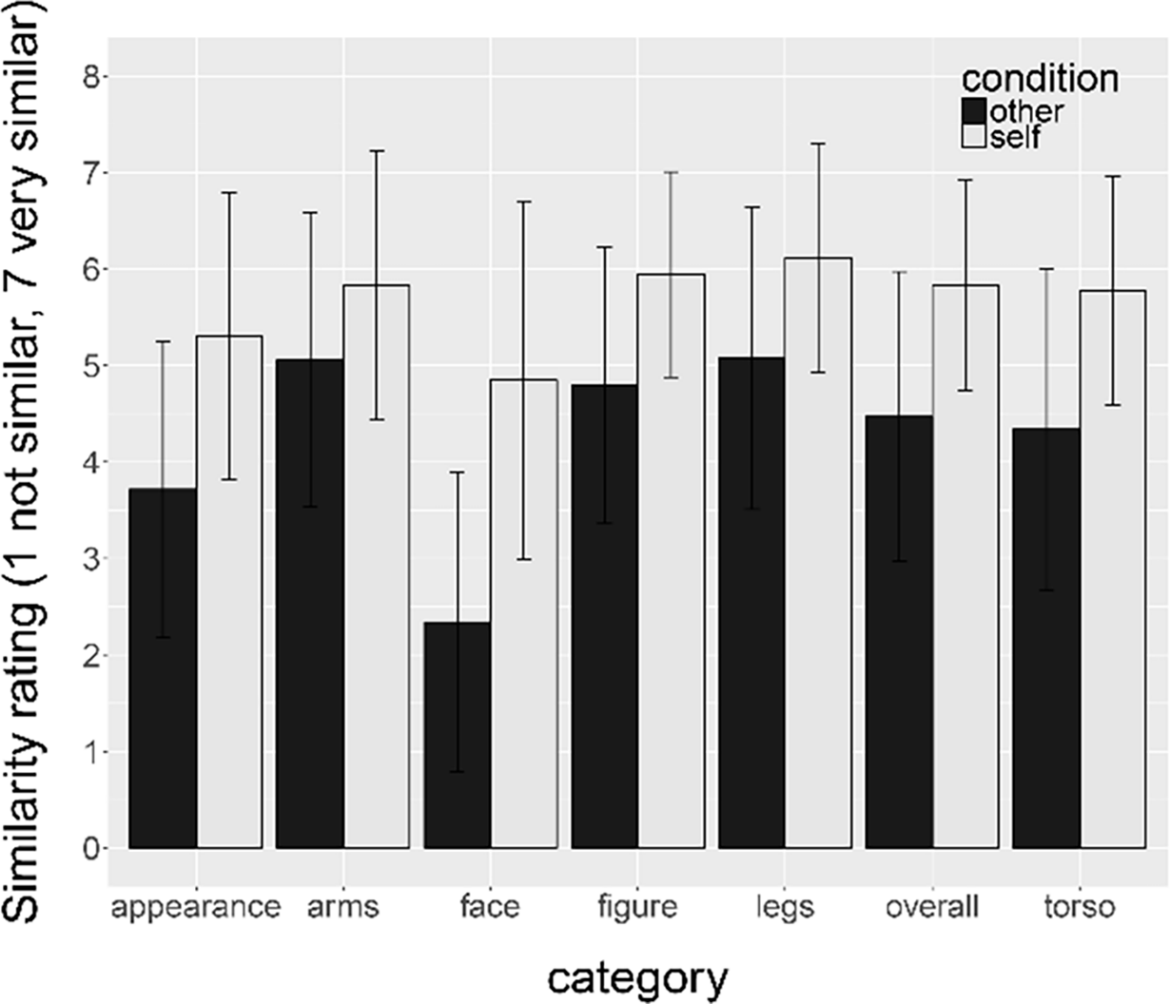
Similarity ratings as assessed by the post-questionnaire for Experiment 1 (own body shape, own texture), Experiment 2a (own body shape, other texture, exposure phase) for all rating categories (appearance, arms, face, figure, legs, overall impression, and torso) for participants that completed all three experiments (n = 53). Error bars represent standard deviation from the mean.

Willingness to accept the avatar as similar to one’s physical body, as indicated by the mean score of the similarity ratings in the post-questionnaire, was regressed onto participants’ BMI to test whether individual BMI had an influence on how similar the avatar was perceived relative to participants’ own body. BMI did not predict the mean score of the similarity ratings (B = .004, *t*(52) = 0.20, *p* = .84, *R*^*2*^ < .01; range: 3.00 to 7.00, *m* = 5.69, *sd* = .96). Participants across the BMI range perceived the personalized avatar equally similar to their physical body. Similarity ratings are shown in Fig 6.

### 3.4 Discussion

The aim of Experiment 1 was to investigate whether personal body size, indexed by BMI, predicts the accuracy of BSE and sensitivity to positive and negative changes in weight relative to the estimated own body size. The results show that participants’ BMI significantly altered the accuracy of estimated own body size in the 1AFC and MoA task, although with small effect sizes (R^2^ = .12 and R^2^ = .17 respectively). Contrary to findings of previous studies where estimates of own body size were biased towards the average body size [26,45], our results show that participants with a BMI in the normal weight und underweight range tended to underestimate their body size, while participants in the overweight and obese weight range tended to overestimate their body size. Further, participants with a higher BMI were less sensitive to changes in body weight of bodies bigger than their estimated own body size as indicated by shallower slopes on the right side of the fitted psychometric function in the 1AFC task. Including a measure of the emotional self-concept, as reflected in the PSY variable, into the analyses did not alter the effect of BMI on the results of the psychophysical experiments, suggesting that the observed effects were either driven by body size per se, or a related factor that did not strongly load onto the PSY variable.

From Experiment 1, it remains an open question as to whether these results would replicate when estimating the size of a body of another identity, or whether they are specific to estimating the size of a body with own identity. In Experiment 2a and 2b, the identity of the presented bodies was manipulated by altering the texture of the avatars (other vs. own), while keeping the same underlying body shape and experimental design.

## 4 Experiment 2a: Body size estimation of a memorized body with another identity

Experiment 2a aimed at investigating whether the effect of participants’ BMI on the results of the psychophysical experiments in Experiment 1 were due to estimating the size of one’s own body or generalize to a body with another identity. The same psychophysical methods and sets of avatars were used as in Experiment 1, with one important exception: participants viewed the set of avatars with their own body shape, but the texture was altered such that the avatar looked like another person. The manipulation of texture was intended to target only identification with the avatar while keeping body shape constant across the experiments. Previous studies have shown that swapping the texture of the body from self to other significantly reduces identification with the avatars [40,46]. Further, by maintaining the body shape information, which is a strong cue used for estimating body weight, we controlled for factors such as individual body shape characteristics and visual experience with the body shape, that would have otherwise been confounding variables when comparing the results of BSE of self (Experiment 1) and other (Experiment 2a). If BMI predicts the accuracy of BSE and the sensitivity to weight changes due to factors associated with own identity, then we expect that participants’ BMI does not predict the size estimation of a body with another identity. However, if the results of Experiment 1 were driven by general biases in BSE, then the same pattern should extend to size estimates of a body with another identity.

### 4.1 Method

#### 4.1.1 Participants

The same participants took part in Experiment 2a as in Experiment 1.

#### 4.1.2 Stimuli and scene

**Fig 7:**
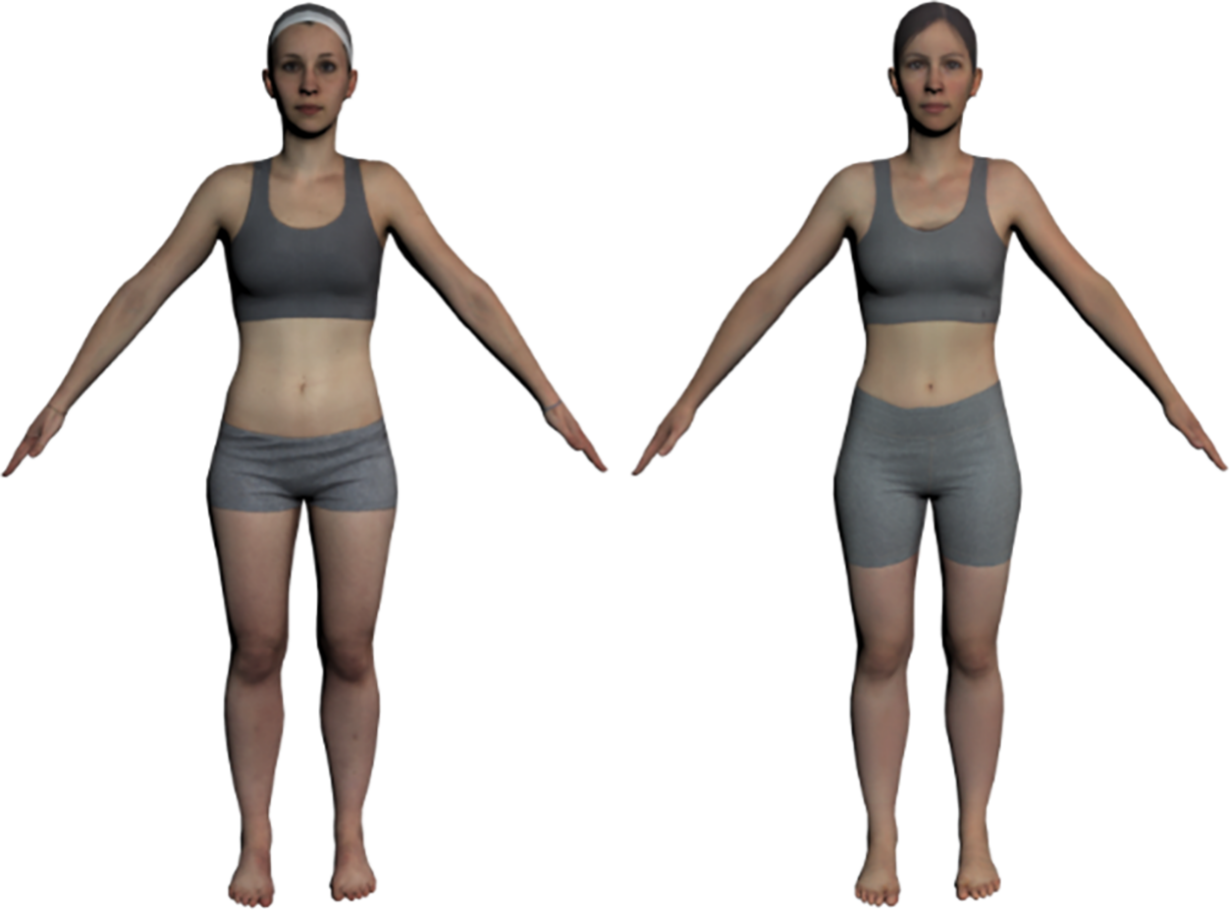
Example of an avatar with own shape and own photo-realistic texture (left, Experiment 1 and 2b) and own shape and photo-realistic texture of another person (right, Experiment 2a).

Participants saw the same set of personalized avatars as in Experiment 1, but with the photo-realistic texture of another person (Fig 7). The texture map was obtained through the same procedure as described in 3.1.3. The same texture was used for all participants, and was matched to the colour of each participant’s photo-realistic texture used in Experiment 1 in Adobe Photoshop CS6.

#### 4.1.3 Procedure

To make sure that the influence of identity (self vs. other) on BSE was not obscured by participants’ expectation to see their personalized virtual avatar generated based on the body scan data, Experiment 2a always followed Experiment 1, separated by a 15 minute break. The same experimental design was used as in Experiment 1, with the exception that the avatar (own shape, other texture) was presented for two minutes before the 1AFC task and again for one minute before the MoA task. Participants were asked to memorize the body shape of the avatar and then respond to the question ‘Is this the correct (memorized) body? Yes/No’ in the 1AFC task. In the MoA task, participants first adjusted the avatars nine times to the size of the memorized body and then nine times to the body size they considered most attractive. To assess how similar each participant thought the to-be-memorized avatar was to her physical body, the same post-questionnaire was used as in Experiment 1.

#### 4.1.4 Statistical analysis

The same analyses were used as in Experiment 1 (see section 3.2). The accuracy of body size estimates was calculated based on the BMI of the memorized body.

### 4.2 Results

#### 4.2.1 1AFC

*Peak*. Estimated ‘correct’ (memorized) body size, in raw units, was regressed onto participants’ BMI to test whether BSE of the memorized body varied based on individual BMI. Participants’ actual BMI did not predict the accuracy of BSE (B = .1, *t*(52) = 1.15, *p* = .25, *R*^*2*^ = .02). On average, participants accurately estimated the memorized body size with a mean overestimation of 0.45% (*sd* = 4.86) (Fig 4, top right), a difference that was not significantly different from 0, *t*(53) = 0.75, *p* = .75.

*Slopes*. There was insufficient data to fit a Weibull function for one participant on the left side. The fit of the psychometric functions to the data of the remaining participants was good (mean *R*^*2*^ = .99, *sd* = .01; left side: mean *R*^*2*^ = 1.00, *sd* = .01; right side: mean *R*^*2*^ = .99, *sd* = .01). Slope steepness (beta) was regressed onto participants’ BMI separately for the left and right slopes to test whether sensitivity to weight changes of the memorized body depended on individual BMI. BMI did not predict slope steepness on the left side (B = -.10, *t*(50) = -1.13, *p* = .26, *R*^*2*^ = .02) or the right side (B = -.22, *t*(51) = -1.393, *p* = .17, *R*^*2*^ = .04) (Fig 4, bottom right).

#### 4.2.2 MoA

Estimated ‘correct’ (memorized) and adjusted most attractive body size, in raw units, were separately regressed onto participants’ BMI to test whether the results varied based on individual BMI (Fig 5, right). Participants’ BMI did not significantly predict BSE of the memorized body (B = 0.01, *t*(52) = -0.13, *p* = .901, *R*^*2*^ < .01). The memorized body size was underestimated on average by 0.66% BMI units (*sd* = 2.87), a difference that was not significantly different from 0, *t*(53) = -1.7, *p* = .10. Participants’ BMI however significantly predicted raw scores of the body size that was perceived most attractive (B = -.65, *t*(52) = -6.7, *p* < .001, *R*^*2*^ = .47), similar to participants’ ideal body size in Experiment 1.

#### 4.2.3 Experimental manipulation check

To investigate whether altering the texture from own (Experiment 1) to other (Experiment 2a) affected perceived similarity between the avatar’s appearance and own physical body, the similarity ratings of Experiment 1 and 2a were compared (see Fig 6). For one participant, the similarity ratings of Experiment 2a were not available; the data of the remaining 53 participants was analysed. A repeated measurement analysis of variance (ANOVA) was conducted with condition (Experiment 1, Experiment 2a) and rating category (overall impression, figure, appearance, arms, legs, torso and face) as within-subject factors. There was a main effect of condition, *F*(1, 52) = 62.16, *p* < .001. Scores were higher when participants where judging their personalized avatar as shown in Experiment 1 (*m* = 5.66, *sd* = 1.4) compared to Experiment 2a (*m* = 4.26, *sd* = 1.77). Thus, the experimental manipulation of altering the identity of the avatar by swapping the body texture from own (Experiment 1) to other (Experiment 2a) made participants to perceive the avatar as less similar to their own physical body. Further, even though some participants reported afterwards they noticed that the avatars in Experiment 2a had some similarities with their set of personalized avatars in Experiment 1, none of them had guessed that the underlying body shapes were identical. The difference in perceived similarity between the avatars in Experiment 1 and 2a was further dependent on the rated feature of the body. Because rating category failed the test of sphericity, the results reported are Greenhouse-Geisser corrected. The ANOVA revealed a main effect of rating category, *F*(3.89, 202.57) = 40.31, *p* < .001. Post hoc paired *t*-tests showed that participants perceived the avatar with the ‘other’ texture to be less similar to their physical body than the avatar with own photo-realistic texture in all seven rating categories (overall impression: *t*(52) = 5.86, *p* < .001; figure: *t*(52) = 5.5, *p* < .001; appearance: *t*(52) = 5.81, *p* < .001; arms: *t*(52) = 3.82, *p* < .001; legs: *t*(52) = 4.54, *p* = .03; torso: *t*(52) = 6.37, *p* < .001; face: *t*(52) = 8.18, *p* < .001). Further, there was a significant interaction between condition and rating category, *F*(4.65, 241.61) = 8.3, *p* < .001. Not surprisingly, the biggest difference in perceived similarity between self (Experiment 1) and other (Experiment 2a) was found in the face, the area that is most informative for the identity of a body.

### 4.3 Discussion

The aim of Experiment 2a was to investigate whether the results from Experiment 1 would replicate when estimating the size of a body with another identity. Contrary to Experiment 1, own BMI did not predict the accuracy of BSE and the sensitivity to changes in weight of a previously memorized body with another identity. This suggests that the effect of personal BMI on BSE is unlikely to arise from general biases in BSE, but rather from factors that are specific to self-identity. However, there was a methodological difference between Experiment 1 and 2a that might explain accurate performance in Experiment 2a, namely participants memorized the body before Experiment 2a. To control for the differences in experimental design, Experiment 2b was conducted where participants memorized their own body beforehand.

## 5 Experiment 2b: Body size estimation of a memorized body with own identity

Experiment 2b investigated whether the difference in results in Experiment 1 and 2a might be due to judging the body size of an avatar with own identity, or due to the exposure phase that was introduced before the psychophysical tasks in Experiment 2a. The same design was used as in Experiment 2a, with the exception that participants saw their own personalized avatar (own shape, own photo-realistic texture) in the exposure phase and were made explicitly aware that this was their body. Participants judged whether the presented bodies corresponded to the memorized body’s size rather than to their physical body size.

### 5.1 Method

#### 5.1.1 Participants

Out of the 54 participants that took part in Experiment 1 and 2a, 26 returned to participate in Experiment 2b. The mean BMI of the 26 participants was 29.19 (*sd* = 7.97; BMI range: 16.94 to 43.89).

#### 5.1.2 Stimuli and scene

Participants saw the same set of personalized avatars (own body shape, own texture) as in Experiment 1.

#### 5.1.3 Procedure

Experiment 2b took place 82.81 days (*sd* = 70.04) after the body scan session, and 60.85 days (*sd* = 65.82) after Experiment 1 and 2a. The design was identical to Experiment 2a, with the exception that participants saw their own personalized avatar (own shape, own texture) for 2 minutes before the 1AFC task and for 1 minute before the MoA task. Participants were told that they would see their own personalized avatar generated based on the body scan data and were asked to memorize the body shape and respond to the question ‘Is this the correct (memorized) body? Yes/No’. After the experiment, participants’ weight was measured and they filled out the same post-questionnaire as in Experiment 1 and 2a to assess subjectively perceived similarity of the avatars to their physical body.

#### 5.1.4 Statistical analysis

The same analyses were used as in Experiment 1 and 2a (see section 3.2).

### 5.2 Results

Participants’ BMI had decreased by 0.24 BMI units (*sd* = 0.77, range: -1.6 to 2.47 BMI units) between the scan session and the experiment. The BMI change was not significantly different from 0, *t*(25) = -1.59, *p* = .12.

#### 5.2.1 1AFC

**Fig 8:**
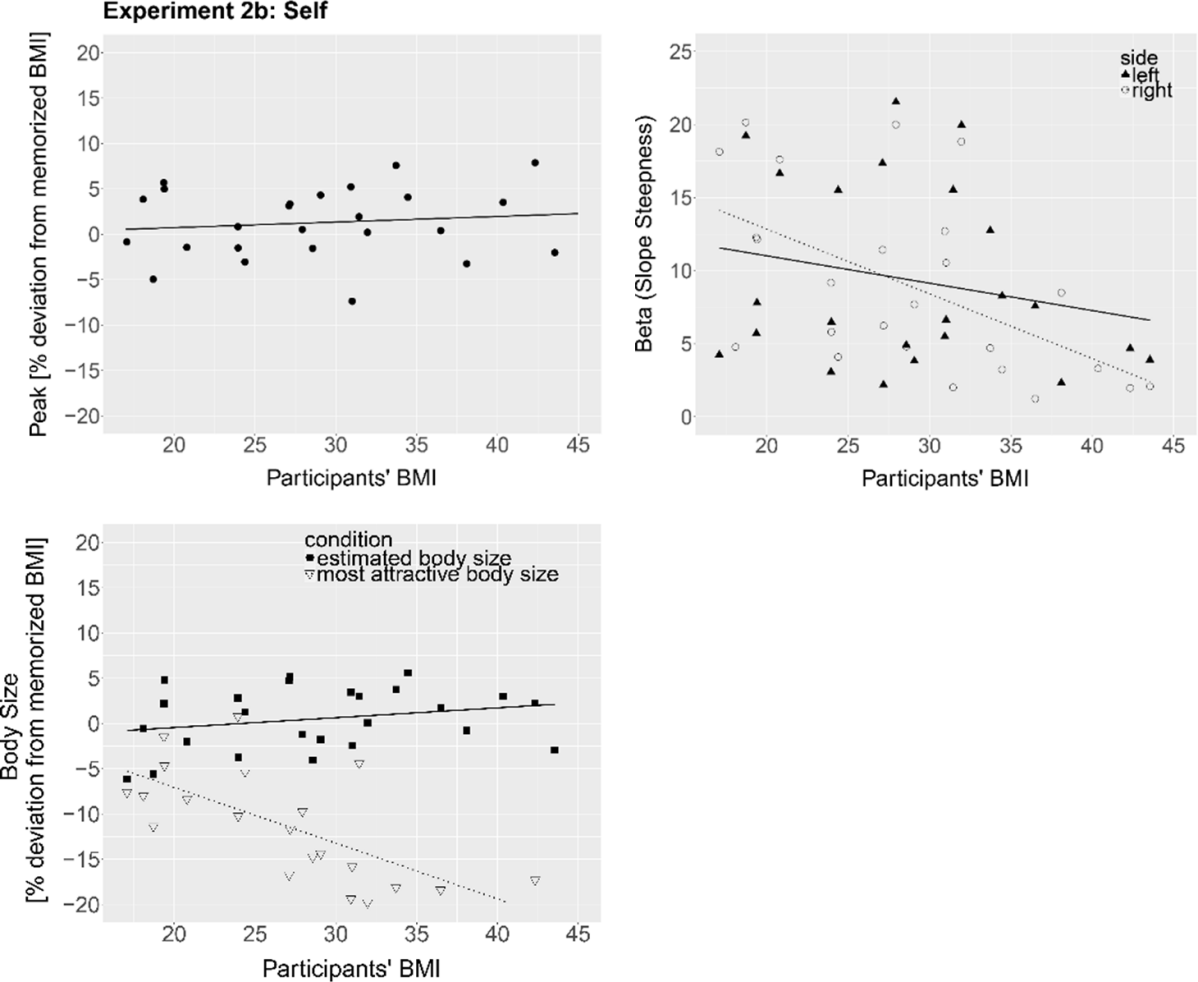
*Top Left*: Percent deviation of the estimated ‘correct’ (memorized own) body size (peak of the psychometric function) from the actual BMI (in %-BMI units) as a function of participants’ BMI in the 1AFC task. The line represents a regression line. *Top Right*: Beta values (slope steepness) for presented bodies smaller (left side of the peak; triangles) and bigger (right side of the peak; circles) than estimated ‘correct’ (memorized) body size as a function of participants’ BMI in the 1AFC task. Higher beta values indicate greater sensitivity to %-BMI changes. The lines indicate the regression lines of beta values on the left side (solid line) and on the right side (dotted line). *Bottom Left*: BMI deviation [in %] of the estimated ‘correct’ (memorized) (squares, solid line) and most attractive body size (triangles, dotted line) as a function of participants’ BMI in the MoA task of Experiment 2b. The lines represent regression lines.

*Peak*. Estimated ‘correct’ (memorized) body size (peak) was regressed onto participants’ BMI to test whether BSE of own previously memorized body varied based on individual BMI. Participants’ BMI at the time of the experiment did not predicted accuracy of BSE of the memorized body (B = .67, *t*(24) = 2.01, *p* = .06, *R*^*2*^ = .14), though it was close to the chosen significance level of .05. On average, participants overestimated the memorized body size by 1.67% BMI units (*sd* = 4.3) (Fig 8, left), a difference that was not different from 0, *t*(25) = 1.97, *p* = .06.

*Slopes*. There was insufficient data to fit a Weibull function for one participant on the right side. The fit of the psychometric functions to the data was good (mean *R*^*2*^ = .998, *sd* = .004; left side: mean *R*^*2*^ = .998, *sd* = .004; right side: mean *R*^*2*^ = .999, *sd* = .003). Slope steepness (beta) was regressed onto participants’ BMI separately for the left and right side. As in Experiment 1, BMI did not predict slope steepness (beta) on the left side (B = -.33, *t*(24) = -0.66, *p* = .51, *R*^*2*^ = .02), but significantly predicted beta on the right side (B = -.69, *t*(23) = -3.20, *p* = .004, *R*^*2*^ = .31). Sensitivity to bodies bigger than the estimated memorized (own) body size significantly decreased as participants’ BMI increased (Fig 8, top right).

Since some participants had changed weight between the scan and Experiment 2b, participants’ actual body weight did not correspond to the memorized body. Although participants were instructed to use the memory of the avatar’s body as a reference, we cannot exclude the possibility that participants used current knowledge of their body and/or online bodily cues for their size estimates. To address this, the linear regression analyses on accuracy of BSE and sensitivity to weight changes were run again where the accuracy of BSE was corrected for weight changes between the scan and Experiment 2b. None of the peak and slopes results changed.

#### 5.2.2 MoA

The mean response of the nine trials for estimated memorized and most attractive body size was regressed onto participants’ BMI to examine whether accuracy of BSE and perceived most attractive body size varied based on individual BMI. BMI did not predict accuracy of BSE of participants’ own memorized avatar (B = .22, *t*(24) = 0.49, *p* = .63, *R*^*2*^ = .01). The memorized body size was on average set to 0.8% (*sd* = 3.73), a difference that was not significantly different from 0, *t*(25) = 1.1, *p* = .28. Participants’ BMI did predict most attractive body size (B = —.97, *t*(23) = -5.74, *p* < .001, *R*^*2*^ = .59), similar to Experiment 2b and own ideal body size in Experiment 1. On average, participants set the most attractive body to -12.24% (*sd* = 6.57) of the BMI of the memorized body and thus of participants’ BMI at the time of the body scan.

### 5.3 Discussion

The aim of Experiment 2b was to investigate whether the difference in results obtained in Experiment 1 and 2a could be due to estimating the size of a body with own identity (Experiment 1) versus estimating the size of a body with another identity (Experiment 2b), or due to the exposure phase before the psychophysical tasks in Experiment 2a. Similar to the results of the 1AFC task in Experiment 1, participants’ BMI significantly predicted the sensitivity to weight changes of bodies bigger than the estimated memorized body size. However, contrary to the results of Experiment 1, participants’ BMI did not predicted the accuracy of BSE in the 1AFC task and the MoA task. Since participants had to memorize the size of a body with their own identity during the exposure phase of Experiment 2b that did not perfectly correspond to their physical body size in case they had changed weight between the scan and Experiment 2b, it is possible that subsequent body size estimates might have been based both on the prior memorized body size intertwined with knowledge about the actual body size. These two possible information sources of the body size might have contributed to accurate estimates of the memorized body size, whereas the sensitivity to weight changes and thus the willingness to accept bodies as corresponding to the own body size might have been driven by factors related to the emotional self-concept and thus be similar to when judgements were based on the physical body size as in Experiment 1. Nevertheless, the results of Experiment 1 and 2b suggest that the effect of BMI on the sensitivity to weight increases only occurs when participants judge the size of a body with their own identity.

## 6 General discussion

Previous literature suggests that a disturbed ability to accurately identify own weight and/or changes in own weight may contribute to overweight [5–7] and further, distortions in estimating own body size might be due to biases that generalize to others’ bodies [26,27]. The current research aimed at investigating whether own body size predicts the accuracy of BSE and the sensitivity to weight changes using a depictive body size estimation task assessing an explicit representation of one’s body. Further, by manipulating the identity of the judged bodies (self vs. other), we systematically investigated whether distortions in BSE were due to general biases in estimating body sizes or were self-specific, and whether they could be explained by cognitive-affective factors as assessed by several validated questionnaires. Our results show that the accuracy of own BSE was indeed predicted by personal BMI in Experiment 1, such that low BMI individuals underestimated their body size and high BMI individuals overestimated their body size. Further, with increasing BMI, participants were less sensitive to weight changes of bodies bigger than their estimated own body size. Crucially, this was only the case when participants estimated the size of a body they identified with (Experiment 1 and 2b), not however when they estimated the size of a memorized body with another identity (Experiment 2a). This suggests, that the effect is most likely due to conceptual factors linked to own identity or emotional self-concept, such as cognitive-affective factors, rather than due to a general bias in estimating body size.

One of the major aims of this study was to investigate whether the ability to identify own weight is predicted by personal body size. Previous studies have suggested that the accuracy of estimated body size of self and others may be influenced by bodies in the environment by means of an average-based reference body, such that the size of low BMI bodies is overestimated and the size of high BMI bodies is underestimated [26,27]. In the present experiments, participants’ BMI predicted the accuracy of BSE such that low BMI individuals underestimated their body size and high BMI individuals overestimated their body size (Experiment 1). Across the BMI range, participants were accurate in estimating the body size of a body with another identity (Experiment 2a). The difference between our results and previous results may be due to the way in which participants were asked to judge their body size. In our study, the question was directed at the participant (‘Is this your body? Yes/No’) while emphasising in the instructions that the question referred to the body’s weight. Previous studies found a contraction bias when participants estimated own body size by judging whether BMI variations of a non-personalized body were *smaller* or *larger* than themselves [26], or when the size of individual body parts had to be adjusted on a personalized body image by moving a slider [24,25]. It is possible, that body size estimates are only biased toward the average body size if the task forces participants to compare own body size to an average-based reference body either because of the use of non-personalized images, or because the size of single body parts has to be adjusted separately. In line with this, when judging the absolute weight of other bodies along the BMI range, size estimates were biased toward the average body weight [27], suggesting that the average body size was used to inform weight judgements. It is up to future research to investigate under which circumstances BSE is biased towards the average body size or influenced by own body size.

Further, BSE might be differently biased when asked to respond to the question ‘Is this your body? Yes/No’ as compared to ‘Is this body thinner/smaller or fatter/larger than you?’ as has been used in many previous studies. Previous research has shown that body size estimates are sensitive to the given instructions [47], and differences have repeatedly been found in patients with anorexia nervosa when instructing them to estimate their body size according to how they *feel* compared to how they *think* they are, with larger size estimates of how they *felt* to be [48–51]. The words *thinner* and *fatter* put emphasis on the appraisal of body weight and might thereby evoke stronger emotions related to own body shape and size. However, when showing personalized body stimuli potentially any question could cause body size estimates to be influenced by feelings towards own body size (e.g. ‘I know/ feel that I am fat’).

Our results show that the sensitivity to weight increases was significantly predicted by participants’ BMI. These results are consistent with findings from previous research showing that participants with obesity were worse in detecting distortions in images depicting their actual body as heavier than in images depicting their actual body as thinner [52,53]. BMI predicted sensitivity to weight changes of bodies bigger than the estimated body size only for self-BSE (Experiment 1 and 2b), not however when estimating the size of a body with another identity but the same body shape (Experiment 2a). It is important to note that our findings indicate a difficulty to detect weight gain that goes beyond the influence of the magnitude of the body size on the ability of detecting difference between bodies, as predicted by Weber’s law [27]. Further, the influence of identity suggests that the lower sensitivity to weight changes of self does not reflect a general bias in estimating body size, but is rather influenced by factors that are specific to self-body judgements and are directly linked to actual body size.

Given the obesity epidemic and the alarming forecast of BMI increase worldwide [1], our results showing that females with overweight and obesity overestimate their body size and have difficulties detecting weight gain have important implications for body weight regulation interventions. In line with the Health Belief Model [4], previous studies have demonstrated that accurate perception of own weight status is strongly associated with efforts to control weight [5,6], and lack of awareness of excess body weight may affect motivation for weight loss [7]. Overestimation of own body size might results from an internalized weight bias that feeds into body dissatisfaction, and this self-stigmatization has been suggested to negatively affect own body image and generally psychological health [54]. Further, weight loss maintenance has been found to present a major challenge [55], and problems in detecting weight gain might be a factor contributing to the difficulty to sustain weight following weight loss. Body weight regulation and maintenance interventions should therefore target the ability to recognize weight changes and to update explicit body representations, including attitudes towards own body size and shape.

Further, ideal body size was lower than estimated own body size for all participants except for two participants with a low BMI. This pattern is in line with previous research showing that women generally wish to be thinner [56,57]. Not surprisingly, several high BMI participants adjusted their desired body size and their subjectively perceived most attractive body weight to the lower end of our range of -20% to +20% of their actual BMI, which for high BMI participants was still an overweight body. Body dissatisfaction is a main psychosocial burden in obesity and has been suggested to be a main factors compromising psychological health [58]. Training programmes should target factors of self-concept related to the own body to improve the evaluation of the own body size and to reduce avoidance behaviour. For example, exposure to one’s body as done in mirror confrontation therapies aimed at improving body image disturbances in eating disorder patients (e.g. [59]), might also be a useful tool for individuals with overweight and obesity.

Finally, a limitation of the present study concerns that the range of body stimuli of -20% to +20% of own BMI was not large enough to accurately assess participants’ desired body size. Future work should therefore consider using a larger range of body stimuli to avoid ceiling effects. Further, the PSY variable that was calculated based on several questionnaires did not comprehensively cover self-concept. With the BSE task that was used in this study, we cannot disentangle whether estimates of own bodily dimensions are based on a body representation that relies on visual information of the body only, or is also influenced by non-visual bodily information. BSE methods have been developed in an attempt to measure the mental representation of people’s body dimensions, often referred to as a component of one’s body image that is traditionally understood as a visual body representation. It is not clear yet whether and how non-visual bodily information influences estimates of own body size in BSE tasks as the one used in this study.

Our study has several important strengths. Using a novel method of personalized 3D avatars that allowed realistic alterations of identity by swapping the body texture, we were able to systematically investigate differences in BSE of self and another identity, while keeping the same underlying body shape. Further, we realistically manipulated the bodies in weight based on a large database of 3D body scans and thereby generated changes in each participant’s body shape that represent their statistically probable body with gained or lost weight. This approach poses a great advantage over studies where variations in body size were created by stretching or compressing photographs of a body since responses to such unrealistic body shape deformations might not truly reflect subjectively perceived body size. Moreover, the bodies were presented in life-size and stereo to mimic a scenario of standing in front of a full-length mirror thereby assessing BSE in an ecologically valid scenario.

## 7 Conclusion

There were three main results reported in the current set of studies. First, the accuracy of BSE is influenced by personal BMI. Participants with higher BMI overestimated their body size, while participants with lower BMI underestimated their body size. Second, sensitivity to changes in body size was predicted by participants’ BMI for bodies bigger than their estimated own body size. Participants with a higher BMI were more willing to accept an even heavier body as their own than participants with a lower BMI. Third, these effects were only apparent when participants estimated the size of a body with their own identity, not when they estimated the size of a body with another identity but the same underlying body shape. The different influence of BMI on BSE for self and other identity suggests that the effect of own BMI on BSE is self-specific and not generalizable to other bodies. Given the increase in BMI worldwide, it is crucial to better understand how own body size can influence the estimation of own body size and the ability to detect weight gain. Training programs using for example avatars matched for certain bodily dimensions in virtual environments could improve the ability to recognize own weight and weight gain.

## Supporting information

S1 File 1AFC results. Results of the accuracy of body size estimation and sensitivity to weight changes in the 1AFC task of Experiment 1, 2a and 2b.
(CSV)
